# Efficacy of high-frequency spinal cord stimulation for fibromyalgia syndrome in two cases: case reports

**DOI:** 10.1186/s40981-023-00660-6

**Published:** 2023-10-21

**Authors:** Mikiko Horita, Ayumi Yasuhira, Mikako Hirakawa, Aisa Watanabe, Nobuhiro Higaki, Tasuku Nishihara, Toshihiro Yorozuya

**Affiliations:** Department of Anesthesia and Perioperative Medicine, Ehime University Graduate School of Medicine, Toon, Ehime 791-0295 Japan

**Keywords:** Fibromyalgia syndrome, Treatment, Spinal cord stimulation, 1000-Hz stimulation, SCS trial

## Abstract

**Background:**

Reports on the effectiveness of spinal cord stimulation (SCS) for the alleviation of fibromyalgia syndrome (FMS) pain are scarce. We report two cases of effective high-frequency SCS at 1000 Hz against upper- and lower-limb pain in patients with FMS.

**Case presentation:**

Two women with widespread pain were diagnosed with FMS and the pain gradually worsened. A 1-week SCS trial was conducted in each patient. In both cases, the patients complained of unpleasant sensations during 10-Hz SCS. However, the pain was alleviated after 1000-Hz stimulation without irritation. Therefore, leads and a generator were implanted, after which they felt almost no pain. Moreover, the dose of the oral medication could be reduced and the patients returned to their daily lives.

**Conclusion:**

SCS at 1000 Hz may effectively treat pain associated with FMS. Therefore, performing an SCS trial for patients with FMS with intractable pain might be worthwhile.

## Background

Fibromyalgia syndrome (FMS) is a form of chronic widespread pain in the classification of chronic primary pain by the International Classification of Diseases 11th revision (ICD-11). This syndrome is defined as diffuse musculoskeletal pain in at least four of five body regions and in at least three or more body quadrants (as defined by the upper–lower/left–right parts of the body) and axial skeleton (neck, back, chest, and abdomen) [1]. It is associated with sleep disorders, cognitive dysfunction, and somatic symptoms. A diagnosis of FMS is established if the pain is not directly attributable to a nociceptive process in these regions and if there are features consistent with nociplastic pain [1].

Patients with FMS present a lower pain threshold, which generates a condition of diffuse hyperalgesia and/or allodynia. This indicates that there may be a problem with the amplification of pain or with sensory processing in the central nervous system (CNS) [2]. Pharmacological treatment alone is inadequate for most patients who suffer from FMS. Considering the different mechanisms of pain sensitivity, multidisciplinary treatment programs are recommended to target the peripheral, central, cognitive–emotional, and interpersonal causes of chronic pain [2].

Spinal cord stimulation (SCS) has been reported to be effective for neuropathic and ischemic pain, as well as central pain [3]; however, few studies have addressed the effectiveness of SCS for FMS pain [4]. Patients with FMS suffer from refractory chronic pain and limited daily activity. As we supposed that SCS might be an option to improve the refractory pain that accompanies FMS, we conducted a 1-week, temporary SCS trial. The pain was alleviated after 1000-Hz stimulation, without irritation; therefore, leads and an internal pulse generator (IPG) were implanted in the patients. We report two cases in which SCS at 1000 Hz was effective against chronic lower-limb pain in patients with FMS.

The authors obtained written informed consent from the patients regarding the publication of this case report and the use of related data.

### Case presentation

#### Case 1

**Fig. 1:**
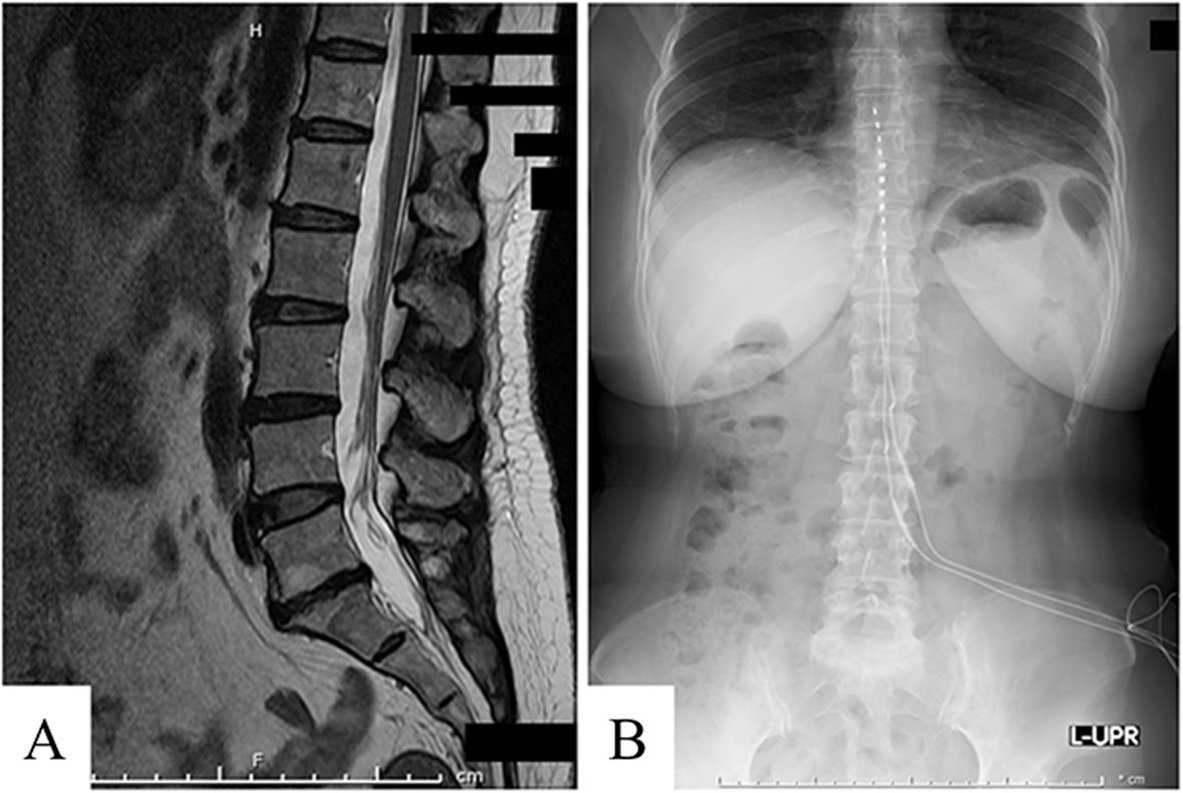
Preoperative and postoperative photographic images of Case 1. **A** Preoperative magnetic resonance image of the lower spine. Spinal canal stenosis was not observed. **B** Abdominal radiograph after SCS placement

A 57-year-old woman who began to feel weakness in her lower legs around the age of 46 and gradually developed generalized pain was diagnosed with FMS by her previous physician. In our hospital, the diagnosis was confirmed by the American College of Rheumatology 1990 Criteria. She was treated with oral medication and physiotherapy, but had difficulty in controlling her pain; thus, she was referred to our department at age 51. The oral administration of pregabalin, duloxetine, neurotropin®, tramadol, and clonazepam helped ameliorate the pain, and the patient was able to continue with her daily life. However, from age 53, the pain in her lower limbs worsened, and she had difficulty in walking. Lumbar spine magnetic resonance imaging revealed a bulge in the lower lumbar spine disks and mild dural sac exclusion, but no significant spinal canal stenosis (Fig. 1A). After providing the patient with sufficient explanation, a 1-week SCS trial for the management of pain in the lower limbs was conducted. During the trial, the patient complained of discomfort from the SCS (10 Hz). However, the pain in the lower limbs was reduced (numerical rating scale [NRS] 6–7 to 1), and the gait difficulty was improved after 1000-Hz stimulation, without irritation. Therefore, SCS implantation (Medtronic® 1000 Hz) was performed (Fig. 1B), after which the patient felt almost no pain in the lower limbs, the dose of oral medication was reduced to only tramadol and duloxetine and could return to her daily life (The final setting: amplitude 3.0 mA, pulse width 60 μs, frequency 1000 Hz).

#### Case 2

**Fig. 2:**
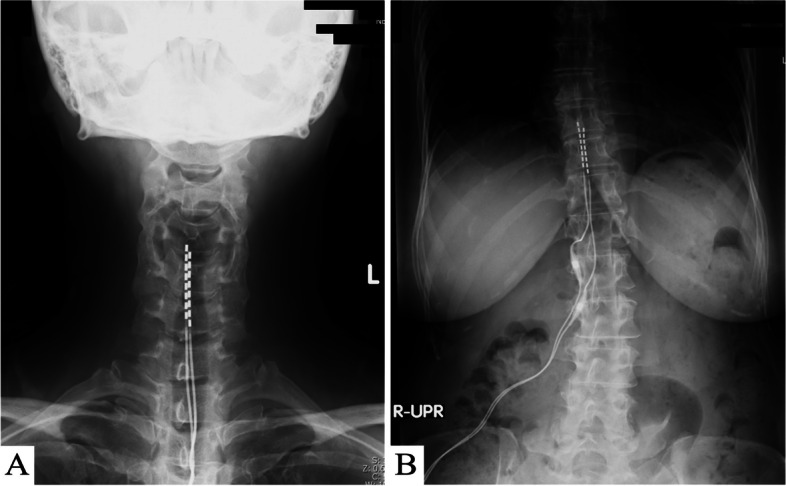
Radiograph of case 2 after SCS placement. **A** Cervical radiograph after SCS implantation. **B** Thoracic radiograph after SCS implantation

The patient was a 64-year-old woman. After a traffic accident at the age of 47, there was an onset of pain in the area from the left cervical region to the arm, as well as discomfort in the lower legs. Radiographic findings revealed a suspected bone injury in the uncinate process of the seventh cervical vertebra. Therefore, a traumatic cervical syndrome was diagnosed, and the patient underwent drug and block therapies such as facet joint block. From around age 49, the pain spread throughout her body, and the patient was diagnosed with FMS by the American College of Rheumatology 1990 Criteria. Treatment with oral medication (gabapentin, tramadol, and pregabalin) and physiotherapy were initiated, and a posterior cervical medial branch block was performed. However, from the age of around 60 years, the pain in the lower limbs worsened, and she had difficulty walking. Because her tolerance toward the oral medication was low and it was challenging to increase the dose so controlling pain could be controlled, a 1-week SCS trial was conducted for the management of pain in the upper and lower limbs. During the trial, 1000-Hz stimulation was attempted because the patient complained that the stimulation (10 Hz) was unpleasant; after SCS, the patient found that the pain in the upper and lower limbs was ameliorated (NRS 6 to 4) and that it became easier to move up and down stairs. Furthermore, the stiffness of the patient’s hands was reduced. At the age of 61 years, SCS implantation was performed for pain relief in the upper and lower limbs (Medtronic® 1000 Hz) (Fig. 2A, B). After implantation, the dose of oral medication was reduced to tramadol taken only once and her gait difficulty was ameliorated (The final setting: amplitude 4.5 mA, pulse width 90 μs, frequency 1000 Hz for both electrodes in the cervical spine and in the lower thoracic spine).

## Discussion

The etiology and pathogenesis of FMS consist of the development of central sensitization and hypersensitivity at the spinal cord and brain levels in the nociceptive neural pathways [1]. Furthermore, it has been indicated that pain hypersensitivity and allodynia are established because of network disorders of pain-transmission pathways, such as hyperexcitability of the ascending pain pathway and functional deterioration of the descending pain inhibitory system [5]. In addition, recent studies have suggested that neuroinflammation of the region extending from the thalamus to the limbic system is involved in the pathophysiology of FMS [6].

SCS is a treatment method that alleviates pain by applying a weak electrical stimulation to the dorsal column of the spinal cord. However, the mechanism underlying its analgesic effect has not been fully elucidated. The following two mechanisms have been considered [7]. Currently, the stimulation of the dorsal column depolarizes the thick myelinated fibers (Aβ fibers), and forward conduction activates the descending pain-inhibiting system of the CNS, resulting in the inhibition of the abnormal activities of the wide dynamic range (WDR) neurons in the dorsal horn. The WDR neurons are inhibited by the segmental activation of the inhibitory intermediate cells in the dorsal horn of the spinal cord via the collateral branch of Aβ fibers by retrograde conduction; alternatively, the transmission within the dorsal roots of Aβ fibers causes peripheral effects. At the same time, the activation of the descending pain-inhibitory system by SCS leads to the attenuation of projection neuron firing in the dorsal horn by modulating the release of neurotransmitters such as glutamate, gamma-aminobutyric acid (GABA), and acetylcholine [3]. As it is said that the descending pain inhibitory system is attenuated in patients with FMS, the analgesic effects of SCS against FMS pain may be due to the attenuation of projection neuron firing in the dorsal horn by activating the descending pain inhibitory system.

High-frequency SCS is considered as paresthesia-free SCS. Though the mechanisms of pain relief with high-frequency SCS remain to be elucidated, three working hypotheses have been presented initially, which are (1) a reversible depolarization blockade, (2) desynchronization of neural signals, and (3) membrane integration. While depolarization blockade and membrane integration offer a reasonable physiologic explanation of SCS relief, there have been demonstrated that direct activation or conduction block of dorsal horn or dorsal root ganglion neurons requires a higher amplitude than the current clinical devices can deliver [3]. Pain relief with high-frequency SCS does not correlate to the territory of paresthesia, supporting a novel mechanism of action. Consistently demonstrated in both preclinical work and clinical experience, the antinociceptive effects of high-frequency SCS inevitably have a delayed onset compared to traditional SCS [3].

Patients with FMS with a low pain threshold perceive even minor stimuli as being unpleasant or painful. They often complain that even 10-Hz SCS is unpleasant, causing them to drop out of SCS trials. However, because 1000-Hz stimulation does not cause irritation or discomfort, the evaluation of the SCS effects in patients with FMS has become possible. In a previous report, SCS at 10 kHz was effective for widespread pain, including FMS [4]. Considering this report and our cases, SCS stimulation over at least 1000 Hz was postulated to be useful for pain relief in FMS. Furthermore, when performing SCS, it is possible to use percutaneous lead insertion (1-week SCS trial), which has the advantage of being minimally invasive, even for pain-sensitive patients with FMS.

Most patients with FMS complain of generalized pain, with 82% reporting arthralgia, 70.9% reporting myalgia, and 42.7% reporting other soft tissue pain, with a high overlap [6]. These spinal diseases may present with clinical symptoms that are inconsistent with the findings, even if there are some abnormalities on imaging, and the degree and extent of pain may be beyond comprehension [8]. Nevertheless, SCS has been shown to be effective in treating several spinal disorders [9], and it may be worth attempting it in patients who are not indicated for surgical treatment but have complaints of severe pain.

## Conclusion

We reported two cases in which high-frequency SCS was indicated for the management of FMS. Although its mechanism of action remains unclear, SCS at 1000 Hz might be an effective treatment for pain associated with FMS and concomitant diseases. It might be worthwhile to perform a SCS trial in patients with FMS with intractable pain.

## Data Availability

All data generated or analyzed during this study are included in this published article.

## Abbreviations

FMS: Fibromyalgia syndrome
SCS: Spinal cord stimulation
NRS: Numerical rating scale
WDR: Wide dynamic range
